# Role of DNA Damage Response in Suppressing Malignant Progression of Chronic Myeloid Leukemia and Polycythemia Vera: Impact of Different Oncogenes

**DOI:** 10.3390/cancers12040903

**Published:** 2020-04-07

**Authors:** Jan Stetka, Jan Gursky, Julie Liñan Velasquez, Renata Mojzikova, Pavla Vyhlidalova, Lucia Vrablova, Jiri Bartek, Vladimir Divoky

**Affiliations:** 1 Department of Biology, Faculty of Medicine and Dentistry, Palacky University, 77515 Olomouc, Czech Republic; stetka.j@gmail.com (J.S.); jan.gursky@upol.cz (J.G.); jlv_15@hotmail.com (J.L.V.); r.mojzikova@gmail.com (R.M.); vyhlidalova.pavla@gmail.com (P.V.); 2 Department of Histology and Embryology, Faculty of Medicine and Dentistry, Palacky University, 77515 Olomouc, Czech Republic; 3 Department of Hemato-Oncology, University Hospital and Faculty of Medicine and Dentistry, Palacky University, 77520 Olomouc, Czech Republic; lucia.vrablova@fnol.cz; 4 Institute of Molecular and Translational Medicine, Faculty of Medicine and Dentistry, Palacky University, 77900 Olomouc, Czech Republic; 5 Genome Integrity Unit, Danish Cancer Society Research Center, DK-2100 Copenhagen, Denmark; 6 Laboratory of Genome Integrity, Institute of Molecular Genetics of the ASCR, 14220 Prague, Czech Republic; 7 Division of Genome Biology, Department of Biochemistry and Biophysics, Science for Life Laboratory, Karolinska Institute, SE-171 77 Stockholm, Sweden

**Keywords:** DNA damage response, chronic myeloid leukemia, polycythemia vera, ATM-Chk2 pathway

## Abstract

Inflammatory and oncogenic signaling, both known to challenge genome stability, are key drivers of *BCR-ABL*-positive chronic myeloid leukemia (CML) and *JAK2* V617F-positive chronic myeloproliferative neoplasms (MPNs). Despite similarities in chronic inflammation and oncogene signaling, major differences in disease course exist. Although BCR-ABL has robust transformation potential, *JAK2* V617F-positive polycythemia vera (PV) is characterized by a long and stable latent phase. These differences reflect increased genomic instability of *BCR-ABL*-positive CML, compared to genome-stable PV with rare cytogenetic abnormalities. Recent studies have implicated BCR-ABL in the development of a "mutator" phenotype fueled by high oxidative damage, deficiencies of DNA repair, and defective ATR-Chk1-dependent genome surveillance, providing a fertile ground for variants compromising the ATM-Chk2-p53 axis protecting chronic phase CML from blast crisis. Conversely, PV cells possess multiple JAK2 V617F-dependent protective mechanisms, which ameliorate replication stress, inflammation-mediated oxidative stress and stress-activated protein kinase signaling, all through up-regulation of RECQL5 helicase, reactive oxygen species buffering system, and DUSP1 actions. These attenuators of genome instability then protect myeloproliferative progenitors from DNA damage and create a barrier preventing cellular stress-associated myelofibrosis. Therefore, a better understanding of BCR-ABL and JAK2 V617F roles in the DNA damage response and disease pathophysiology can help to identify potential dependencies exploitable for therapeutic interventions.

## 1. Introduction

Chronic myeloproliferative neoplasms (MPNs) are clonal hematopoietic stem cell (HSC) disorders characterized by abnormal proliferation of one or more myeloid lineages. Chronic myeloid leukemia (CML), a Philadelphia chromosome-positive (Ph^+^) chronic MPN, is characterized by the presence of *BCR-ABL* oncogene [1]. Philadelphia chromosome-negative (Ph¯) MPNs encompass a spectrum of clonal hematological disorders, which include three main clinical entities: polycythemia vera (PV), essential thrombocythemia (ET), and primary myelofibrosis (PMF) [2]. PV is predominantly associated with oncogenic V617F mutation in the *JAK2* gene, detected in more than 95% of cases diagnosed [3,4,5,6]. All MPNs are characterized by chronic inflammatory state (reviewed in [7,8,9]). Thus, oncogenic and inflammatory signaling, both known to fuel genotoxic stress and tumorigenesis in the hematopoietic system in a cell-autonomous and non-cell-autonomous manner, converge in disease evolution of MPNs [10,11,12,13,14]. Therefore, CML and PV provide an excellent model of inflammation-associated neoplasia for investigating mechanisms of DNA damage accumulation and DNA damage response (DDR) activation throughout early pre-cancerous ontogeny.

Apart from a well-established function of the DDR as the intrinsic biological barrier against activated oncogenes and progression of early stages of solid tumors into overt cancer [15,16,17,18,19], the role of the DDR machinery in the development of myeloid neoplasms and acute myeloid leukemias (AML) is being elucidated relatively recently. Indeed, multiple studies have provided evidence on progression of CML and MPN to fully transformed leukemias by selection for mutations in *TP53* or other major DDR components [20,21,22], but the detailed hierarchical nature of cooperation between the DDR and inflammatory cytokine network in leukemogenesis has remained poorly understood. We described the DDR checkpoint as a critical mechanism rate-limiting for malignant transformation induced by the mixed lineage leukemia (MLL) oncogenic fusion. *Mll-ENL* oncogene synergized with inflammatory factors to trigger checkpoint signaling and senescence, thereby counteracting leukemogenesis in a mouse model mimicking human AML [23]. The nature of intrinsic and extrinsic mechanisms that alter the DDR during the leukemogenic process of AML development has been recently reviewed by Esposito and So [24] and Nilles and Fahrenkrog [25].

The aim of this review is to discuss the emerging role of DDR alterations in the pathophysiology of two chronic myeloproliferative disease states, *BCR-ABL*-positive CML and *JAK2* V617F-positive PV. We highlight similarities and differences in the DDR landscapes of *BCR-ABL*- and *JAK2* V617F-mutated hematopoietic progenitors, the understanding of which is crucial for therapeutic targeting of these diseases, including synthetic lethality approaches.

## 2. Role of DDR in CML and PV

The shared and diverse phenotypic characteristics of chronic MPNs have been attributed to dysregulated signal transduction, a consequence of acquired disease-causing oncogenic mutations, *BCR-ABL* in CML, *JAK2* V617F in Ph^−^ MPNs, and several less common oncogenes found in these diseases [26]. Despite similarities in downstream signaling of BCR-ABL and JAK2 V617F, involving the essential role of STAT5 in induction of myeloproliferative malignancy induced by both oncogenes [27,28,29], major differences between the cellular responses triggered by *BCR-ABL* and *JAK2* V617F oncogenes exist. The chimeric BCR-ABL protein is a constitutively active tyrosine kinase [30,31] that shows a robust transformation potential associated with multiple signaling pathways deregulated or activated by BCR-ABL, such as RAS-mitogen-activated protein kinase (MAPK) and phosphoinositide 3-kinase/Akt pathways [32,33] (reviewed in Ren et al. [34] and Chen et al. [35]). If untreated, the chronic myeloproliferation driven by the BCR-ABL oncogene rapidly progresses to an accelerated phase and terminal blast crisis (BC). In PV, on the other hand, the gain-of-function mutation in the *JAK2* gene (*JAK2* V617F) constitutively activates type-1 myeloid cytokine receptor-mediated signaling [3,4,5,6,36], resulting in myeloproliferation and systemic inflammation with a protracted clinical course and near-normal life expectancy [37]. These differences in progression of CML and PV suggest distinctions in the nature of *BCR-ABL* and *JAK2* V617F oncogene-induced intrinsic and extrinsic mechanisms that govern the myeloproliferation process and its acceleration, including the rate of endogenous DNA damage, DNA damage checkpoint activation, and the extent of genomic instability.

### 2.1. Role of DDR in Chronic Phase of CML and Progression to Blast Crisis

**Figure 1:**
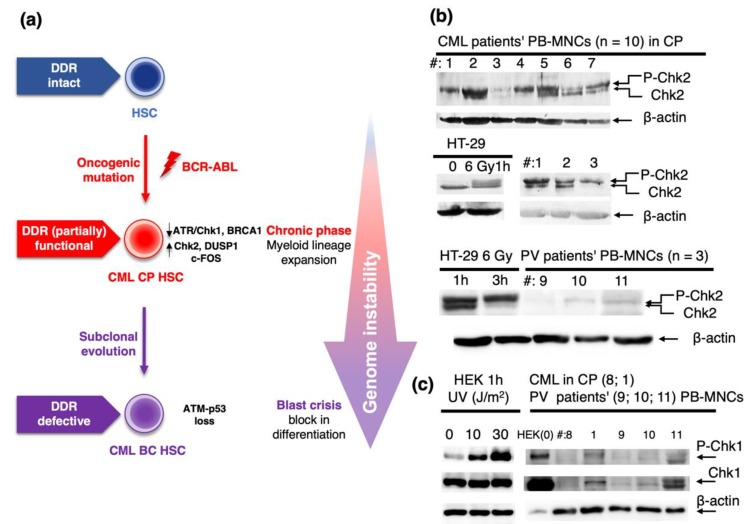
(**a**) Chronic phase (CP) chronic myeloid leukemia (CML) cells expressing the BCR-ABL oncogenic fusion show partially functional DDR, despite reduced activation of the ATR-Chk1 axis causing failure of genome surveillance and increased genome instability. The DDR is marked by activation of Chk2 and the cells exhibit non-oncogenic addiction to c-FOS and DUSPl expression. Inactivation of *TP53* or silencing of Atm signaling leads to fully compromised DDR, allowing acceleration of the disease course to full-blown blast crisis (BC) of CML. (**b**) Expression and mobility shift of Chk2 were determined by immunoblotting analysis of lysates from seven CML and three polycythemia vera (PV) patients (see Supplementary Table S1 for patients’ numbering and details). HT-29 cells non-irradiated (0) or irradiated with a defined dose of gamma irradiation (6 Gy) and harvested after 1 or 3 h after irradiation were used as a positive control for Chk2 activation. For methodology, see the Appendix A. Upper panels: lysates from seven CML patients in CP; three patients were assayed twice in two different assays. Bottom panel: lysates from three PV patients. (**c**) Levels of Chk1 phosphorylation at S345 (P-Chk1) and total Chk1 expression in lysates from two CML and three PV patients. HEK cells untreated or UV-treated with a defined dose of radiation (J/m^2^) were used as a positive control for Chk1 activation. In the patients’ blot, the control HEK cell sample loading was intentionally decreased (compared the β-actin signals) to prevent over-saturated signal on a gel with clinical samples achieving the limit of detection. Patient no. 8 was a CML patient in complete molecular remission after imatinib treatment; no. 1 was a newly diagnosed untreated CML patient. No. 9 was an untreated PV patient, nos. 10 and 11 were PV patients on interferon-α treatment. See also Supplementary Table S1 and Appendix A for details. DDR, DNA damage response; HSC, hematopoietic stem cell; LSC, leukemia stem cell; PB-MNCs, peripheral blood mononuclear cells.

CML is characterized by an indolent, chronic phase (CP) preceding an acute transformation to BC. Failure of DNA damage repair and loss or malfunction of DDR components accompanied by accumulation of DNA damage and genomic instability has been considered in CML evolution [38,39]. It was proposed that *BCR-ABL*-expressing cells feature reduced activation of the ATR-Chk1-mediated DDR signaling, with ensuing accumulation of substantial genomic instability due to replication stress and oxidative damage. Mechanistically, such disruption of ATR-dependent signaling was attributed to nuclear import of BCR-ABL after DNA damage and its binding to ATR [40]. However, contrasting data were also reported, showing that BCR-ABL does activate ATR-Chk1 signaling, reflecting responsiveness of *BCR-ABL*-positive myeloid cells to DNA damage following genotoxic treatment [41]. Some studies also addressed functionality of the ATM-Chk2 signaling axis in CML. Thus, c-Abl is a nuclear tyrosine kinase activated by DNA damage in an ATM-dependent manner [42,43]. Even though the BCR-ABL is predominantly localized to the cytoplasm [44], the aforementioned ability of BCR-ABL translocation to the nucleus after DNA damage led to a proposal that BCR-ABL and c-Abl share multiple protein interactions including that with ATM [40]. As a consequence, ATM-mediated activatory phosphorylation of Chk2 was detected in *BCR-ABL*-expressing cellular models and patient’s cells [40]. These results suggested that ATM-mediated signaling is operational and activated in CP-CML [20], implying that the ATM/Chk2/p53 checkpoint signaling induced by the *BCR-ABL* oncogene may protect CP-CML cells against the blast crisis. Indeed, inactivating mutations of *TP53* were found in up to 30% of cases of BC-CML (reviewed in [20]) and loss of one Atm allele was sufficient for acceleration of the BC in a BCR-ABL transgenic mouse model of CML [45]. Therefore, the ATR-Chk1 and ATM-Chk2 DDR pathways play crucial roles in determination of susceptibility to BC in CML (Figure 1a).

The activation status of Chk2, documented by phosphorylation of Chk2, can be detected by an electrophoretic mobility shift of the Chk2-specific band [46], and multiple phosphorylation sites contribute to this phenomenon [47]. In our yet unpublished study, we analyzed Chk2 activation in peripheral blood mononuclear cells (PB-MNCs) isolated from several CML patients in the CP. The Chk2 kinase was activated in all samples, and the phosphorylation-specific band appeared to be strong in most patients (Figure 1b). These data are consistent with recently published evidence for ongoing DNA damage and DDR detected by activated ATM, Chk2, and γH2AX accumulation in PB-MNCs of de novo untreated CP-CML patients [39]. In contrast to strong Chk2 activation in CML cells, lysates prepared from cells obtained from PV patients in their proliferative phase showed only modest evidence of activated Chk2 (i.e., displayed lower signal of phosphorylated Chk2 compared to CML cells), whereas the unphosphorylated form of Chk2 was barely detectable in these samples (Figure 1b). These data indicated an overall lower extent of Chk2 expression and activation in PV cells compared to CP-CML cells. In addition, we confirmed the published data reporting rather low Chk1 expression and phosphorylation at S345 (P-Chk1) in CP-CML cells; nonetheless, comparably low levels of P-Chk1 were observed in lysates from PV patients (Figure 1c).

Although the ATM-Chk2-p53 pathway seems to be activated in CML and protects CP-CML cells from progression to BC, some additional key components of the DDR machinery, such as the BRCA1 tumor suppressor, are downregulated in CML [48,49]. *BRCA1* is a major DNA repair gene as it promotes homologous recombination (HR) and replication fork protection, playing critical roles in preserving genomic integrity [50]. Another key component of the HR repair of DNA double strand breaks, RAD51, is upregulated in CML, and BCR-ABL has been shown to boost RAD51’s activity through several mechanisms [51]. Furthermore, expression of DNA-dependent protein kinase, catalytic subunit (DNA-PKcs) was shown to be downregulated in CML [52]. DNA-PKcs is an essential part of DNA-dependent protein kinase complex, playing a critical role in DNA double-strand break (DSB) repair and V(d)J recombination. These BCR-ABL-induced effects lead to deregulated HR activity and DNA repair defects, forcing *BCR-ABL*-expressing cells to rely on unfaithful DSB repair pathways [53,54,55,56,57,58], resulting in disruption of overall genome integrity maintenance. Therefore, these features support a conclusion that BCR-ABL induces a mutator phenotype [59]. Evidence that BRCA/DNA-PK-deficient CML leukemia stem cells are highly sensitive to inhibitors of poly-(ADP-ribose) polymerase 1 (PARP1) [60] brought about a potential of targeting key DNA repair enzymes in CML, which are in synthetic lethal relationship with BCR-ABL.

The mutator phenotype of *BCR-ABL*-expressing cells has been also attributed to increased reactive oxygen species (ROS) production. BCR-ABL not only enhances ROS production [55,61,62], but it also promotes oxidative stress in CML cells by repressing antioxidant defenses [63]. Elevated ROS are known to modulate activities of signaling pathways involved in malignant proliferation and apoptosis, such as phosphoinositide 3-kinase/protein kinase B (PI3K/PKB) and mitogen-activated protein kinase (MAPK) signaling pathways through oxidation of negative feedback loop regulators [64,65,66]. Elevated ROS in *BCR-ABL*-expressing cells were found to activate PI3k/Akt pathway via inhibition of protein phosphatase 1 α (PP1α) [67].

The BCR-ABL oncoprotein constitutively activates signaling pathways, which under physiological conditions mediate cellular responses to cytokines. Therefore, BCR-ABL oncogenic signaling and growth factor signaling converge to induce the expression of multiple signaling proteins [35]. Kesarwani et al. [68] showed that two such molecules, c-Fos and Dusp1, are overexpressed in CML and constitute non-oncogene addiction in *BCR-ABL*-induced leukemia. DUSP1 is a member of dual-specificity MAP kinase (MAPK) phosphatases (DUSPs), negative regulators of MAPK signaling in mammalian cells [69]. DUSP1 particularly dephosphorylates stress-activated protein kinase (SAPKs) members of the MAPK superfamily, which include Jun kinases (JNKs) and p38MAPK. The latter kinases are important mediators of DNA damage and inflammatory responses [70]. DUSP1 activity plays a pivotal role in supporting cancer cell survival by buffering SAPK activities under tumor-associated inflammatory conditions [71]. In a CML mouse model, inhibition of Dusp1 activated p38MAPK and sensitized *BCR-ABL*-positive CML stem cells to imatinib with complete clearance of minimal residual disease [68]. Thus, DUSP1 inhibition is synthetically lethal with BCR-ABL and thus may represent a therapeutic approach for CML.

In conclusion, the oncogenic BCR-ABL activation results in DNA damage, but despite proposed partial reduction of ATR-Chk1 activity, the ATM-Chk2-mediated signaling to p53 and other DDR effectors appears to respond to threshold of genotoxic insults, providing a checkpoint barrier against transformation into BC. The survival of CML cells with damaged DNA has been attributed to inhibition of the Bcl-x(L) deamidation pathway, a mechanism that prevents apoptosis in the presence of high DDR [72]. Supra-threshold amounts of DNA damage and genomic instability that occur as a consequence of BCR-ABL cell autonomous and non-cell-autonomous functions during the course of the disease eventually fuel tumor suppressor barrier inactivation (through selecting for p53 mutations and experimental Atm loss, for example), with the subsequent accelerated progression of untreated CP-CML towards the blast crisis.

### 2.2. Role of DDR in Polycythemia Vera

As mentioned, PV have a long clinical course and near-normal life expectancy [37]. Despite conditions of systemic inflammation, the chronic proliferation of *JAK2* V617F-positive PV is sustained over decades with relatively low cumulative incidence of blast transformation (to AML) and fibrotic progression to post-PV myelofibrosis (post-PV MF, [22,73]). On the contrary, an analogous inflammatory microenvironment triggers oxidative stress and accumulation of damaged DNA, which together with oncogene-induced replication stress causes genomic instability and malignant progression in other inflammation-associated myeloid malignancies such as CML, AML, and myelodysplastic syndrome (MDS) [62,74]. Indeed, some studies have described JAK2 V617F-dependent accumulation of DSBs [75], increased oxidative DNA damage [76], impaired HR-mediated DSB repair contributing to genomic instability [77], and increased replication fork stalling that provides a potential source of DSBs [78], subsequently leading to a mutator phenotype. However, some of these datasets are in contrast to long-lasting follow-ups of PV patients, who show sustained genome stability with rare occurrence of cytogenetic abnormalities [73,79]. A possible explanation for this discrepancy is that the experimental data obtained from in vitro cultures and mouse models may not be easily transferable to in vivo oncogene behavior in human patients. Particularly in the case of ex vivo-cultured PV patient-derived CD34^+^ progenitors, one should take into consideration that the hyper-recombination phenotype, observed by Plo et al. [77], was manifested in cells maintained for several days in medium containing DNA damage-promoting cytokines (such as IL-6 [80]) or other growth factors (SCF, IL-3, TPO) promoting ROS production in stimulated human hematopoietic cells [81]. In this regard, we have previously documented that DNA damage protection of *JAK2* V617F^+^ cells is effective under conditions of the relatively modest inflammation-induced degree of DNA damage. These adaptive intrinsic mechanisms are capable of “buffering” the potential genotoxic impact of the cell autonomous and microenvironment-dependent inflammatory stress in PV. However, such a delicate balance can be experimentally altered by enhanced DNA damage and robust activation of the DDR checkpoint response in *JAK2* V617F^+^ cells, higher than that in the *JAK2* wild-type (wt) cells [82].

Even though inflammation contributes to the disease transformation towards post-PV MF with increased risk of neoplastic transformation, the most decisive factor that determines MPN progression is acquisition of additional mutations in genes associated with myeloid neoplasms [83,84,85]. Additional mutations acquired usually in genes encoding epigenome modifiers, such as *DNMT3A*, *TET2,* or *EZH2*, that are also shared between MDS and AML patients, have a potential to rewire the biology towards pre-leukemic-like clones [86]. Thus, compared to early leukemogenesis, the disease evolutionary trade-off—bypass of the DNA damage-checkpoint and hence un-opposed proliferation at the expense of an increased genomic instability—seems to be largely avoided in PV.

**Figure 2:**
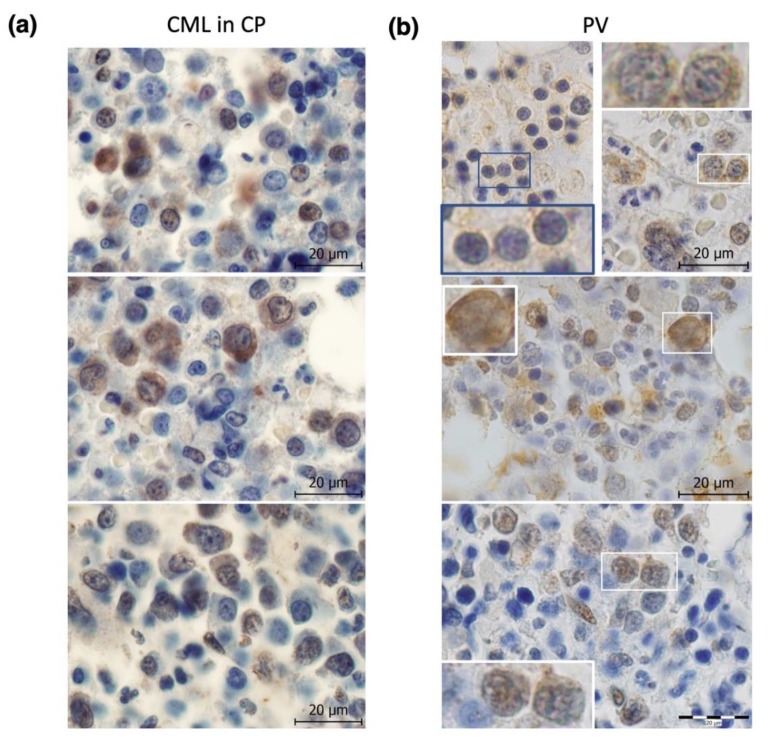
Immunohistochemistry (IHC) staining for ATM phosphorylation at S1981 (P-ATM) in chronic phase (CP) CML (**a**) and PV (**b**) bone marrow trephine biopsies. (**a**) Upper panel: nuclear staining and middle panel: nuclear and cytoplasmic staining in CML patient no. 15; bottom panel: predominantly nuclear and rare nuclear and cytoplasmic staining in CML patient no. 16. Overall, CML cells show numerous nuclear brightly stained P-ATM foci and variable degree of cytoplasmic P-ATM positivity. (**b**) Upper panel: PV (#12) with mostly weak but constantly present cytoplasmic staining (details corresponding to blue lined inset on the left and to white lined inset on the right photographs); middle panel: PV with light fibrosis (#13) with cytoplasmic and nuclear positivity; bottom panel: post-PV MF (#14) revealed only nuclear foci staining. See Supplementary Table S1 for patients’ numbering and details. Scale bars, 20 μm. IHC staining was performed as described [82].

Our own early (unpublished) analysis depicted in Figure 1b revealed differences between Chk2 activation in CML and PV, suggesting differences in functions of its upstream regulator ATM kinase. Although the activated, auto-phosphorylated form of ATM (P-ATM) localizes to the nucleus in bone marrow progenitors of myeloid malignancies including MDS [87,88] and CML ([45] and Figure 2a), our immunohistochemistry (IHC) staining against P-ATM at S1981 revealed predominantly cytoplasmic P-ATM immunoreactivity in PV progenitors and distinct nuclear staining (and lack of cytoplasmic staining) only in post-PV MF ([82] and Figure 2b). These data indicate that although ATM in CML (and MDS and post-PV MF) progenitors regulate its downstream substrates and cell cycle checkpoints mainly from the nucleus, this is different in PV progenitors, where ATM exerts its actions mainly from the cytoplasm. Cytoplasmic activation of ATM in PV likely reflects its response to ROS generated in PV bone marrow inflammatory microenvironment, as ATM phosphorylation by ROS requires its cytoplasmic localization [89]. In addition, the aforementioned extent of Chk2 activation in CML (high) and PV (low) likely reflects subcellular localization of P-ATM [90,91]. Our further (published in [82]) IHC staining of patients’ bone marrow sections from PV revealed low nuclear staining for activated ATR (P-ATR at T1989), and barely detectable expression of a marker for oxidative DNA lesions 8-oxoguanine (8-oxoG), as well as very low staining for a marker of global nuclear DDR activation, Ser 139-phosphorylated histone H2AX (γH2AX) [82]. These data suggested that despite inflammatory microenvironment and *JAK2* V617F oncogene-driven myeloproliferation, certain mechanisms must mitigate the potential genotoxic impact of the overall oncogenic program controlled by *JAK2* V617F, thereby allowing for PV chronic proliferation with relatively stable genome. Importantly, a gradual increase in the nuclear P-ATM (Figure 2b) and γ-H2AX levels [82] following the progression of PV to post-PV MF implied that such buildup of the DDR threshold signaling is linked to suppression of proliferation and fibrogenesis.

According to a recent study, the factor that maintains fork stability of *JAK2* V617F-expressing cells in the proliferative phase of MPN is a DNA helicase RECQL5 [92]. Increased RECQL5 expression protected *JAK2* V617F-positive erythroblasts from DSB formation and cell death through increased single-stranded annealing-mediated DNA repair, thus contributing to genomic stability. *RECQL5* was shown to be a transcriptional target of JAK2 V617F/STAT5, and its knockdown sensitized *JAK2* V617F-expressing cells to hydroxyurea [92].

**Figure 3:**
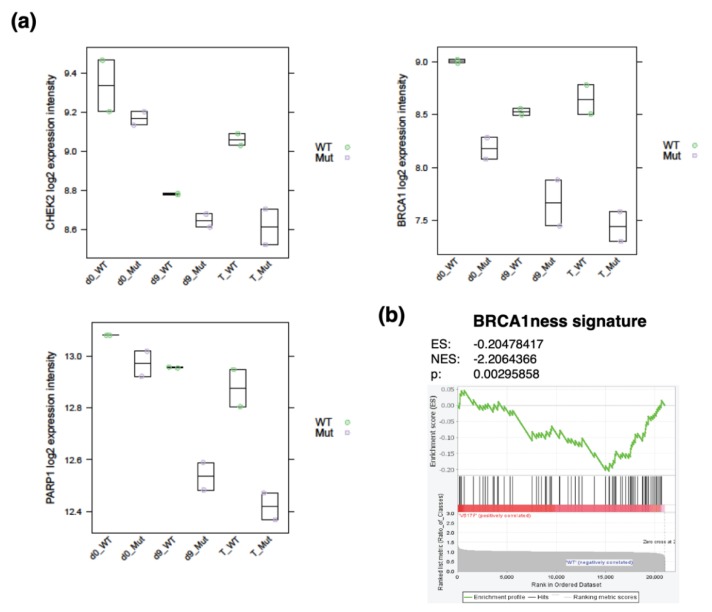
Comparison of *CHK2* and BRCA1-associated DDR gene expression in induced pluripotent stem cell-derived CD34^+^ progenitor-enriched cultures (P-ECs) from a *JAK2* V617F^+^ PV patient and from a *JAK2* wild-type (wt) healthy control [82]. (**a**) *CHK2*, *BRCA1,* and poly-(ADP-ribose) polymerase 1 (*PARP1*) mRNA expression in day 0 (d0) of undifferentiated and day 9 (d9) differentiated *JAK2* wt (WT) and *JAK2* V617F^+^ (Mut) P-ECs, either untreated or treated (T) with inflammatory cytokines IFNγ, TNFα, and TGFβ1 for 24 hours. Two biological replicates per group were used. The plots are based on published gene sets and methodology [82] and linked ArrayExpress database (E-MTAB-7693). (**b**) Gene set enrichment analysis plot of BRCA1ness signature gene set members ([93]; *n* = 77) in day 9 of differentiation of *JAK2* V617F^+^ compared to *JAK2* wt CD34^+^ P-ECs. NES, normalized enrichment score.

In our recent publication, we further elaborated on the concept of protection mechanisms that guard myeloproliferative progenitors from cell-intrinsic and cell-extrinsic DNA damage and thus DDR, facilitating creation of a barrier preventing cell cycle arrest, myelofibrosis, and rapid malignant transformation in *JAK2* V617F-positive PV. We used induced pluripotent stem cell (iPSC)-derived CD34^+^ progenitor-enriched cultures (CD34^+^ P-ECs) from a *JAK2* V617F-positive PV patient and from a *JAK2* wild-type healthy control [82]. The CD34^+^ P-ECs were cultured in the absence or presence of IFNγ, TNFα, and TGFβ1, in order to mimic the PV patients’ microenvironment. The *JAK2* V617F^+^ hematopoietic progenitors treated with inflammatory cytokines had slightly increased but tightly controlled ROS levels when compared to their *JAK2* wild-type counterparts, and exhibited upregulated expression and activity of key enzymes involved in the ROS buffering system. These progenitors were also less prone to accumulate the 8-oxoG oxidative DNA lesions and the γH2AX DDR activity marker, and their overall modest degree of DDR signaling was compatible with ongoing proliferation. Many markers of DDR were downregulated in inflammatory cytokine-treated *JAK2* V617F^+^ CD34^+^ P-ECs compared to *JAK2* wild-type progenitors, including DNA damage-induced repair gene sets ([82], Figure 3a), suggesting protection mechanisms (such as the above-mentioned RECQL5 or DUSP1, mentioned below) of *JAK2* V617F^+^ PV progenitors against DNA-damaging stimuli, and hence lower demand for enhanced expression of most DDR factors. We did not find any defect in HR in *JAK2* V617F^+^ CD34^+^ P-ECs [82] and, in fact, the entire BRCA1-associated DNA repair gene set (BRCA1ness, [93]) was significantly enriched for differentially downregulated genes (Figure 3b). Interestingly, *BRCA1* downregulation was accompanied by decreased *PARP1* expression (Figure 3a). This *BRCA1* downregulation does not seem to be dependent on different fractions of *JAK2* V617F^+^ and *JAK2* wild-type progenitors in individual cell cycle stages [82], but rather it seems to be an inherent feature of proliferating *JAK2* V617F^+^ PV progenitors, similarly to BRCA1 deficiency in *BCR-ABL*-proliferating CP-CML [60]. However, in contrast to PV progenitors and to a fraction of AML and MDS samples with downregulated expression of genes in BRCA1 pathway [60], BCR-ABL causes downregulation of BRCA1 protein [60,94].

**Figure 4:**
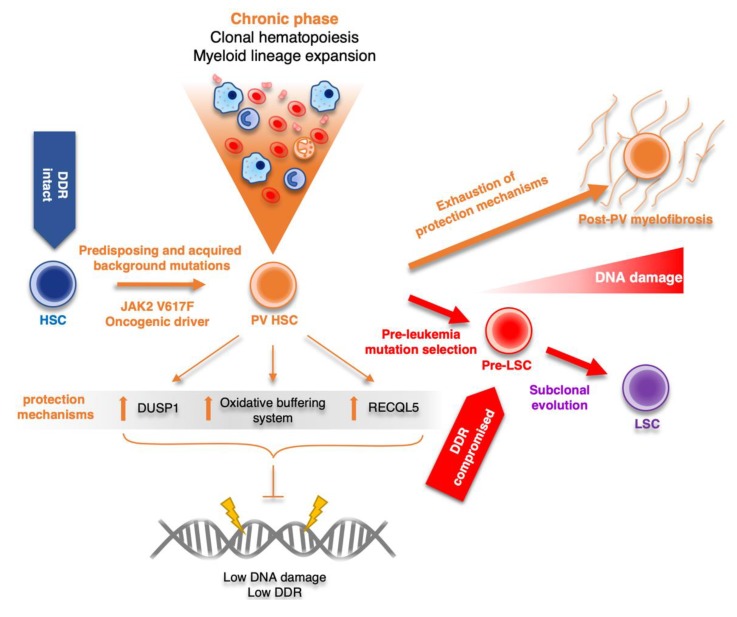
Schematic model of PV disease evolution, showing the central role of JAK2 V617F-dependent protection mechanisms (upregulation of DUSP1, RECQL5, and oxidative buffering system activity) in the overall long-term maintenance of low DNA damage and genomic stability during the chronic proliferation phase of the disease. Upon exhaustion of protection mechanism capacity, the disease progresses mostly towards post-PV myelofibrosis. In a minor subset of cases, selection of pre-leukemia mutations inhibiting DDR and subclonal evolution towards leukemia stem cell (LSC) marks the onset of secondary acute myeloid leukemias (AML). DDR, DNA damage response; HSC, hematopoietic stem cell; Pre-LSC, pre-leukemia stem cell; LSC, leukemia stem cell.

Stimulation of p38MAPK-mediated signaling in chronic inflammatory conditions, such as in PV, would impair proliferation and direct the choice of cell fate towards apoptosis or senescence [95,96], cellular conditions resembling the status of fibrotic bone marrow. Indeed, excessive activation of the p38–MAPK cascade was shown to be associated with PMF [97]. However, the fate of myeloid progenitors in indolent, proliferative PV is at odds with increased SAPK activity. We have shown up-regulation of DUSPs, negative inhibitors of SAPKs, in hematopoietic progenitors derived from PV-patient specific iPSCs, with enrichment of those with substrate specificity for p38 and JNK [82]. We have also detected high expression of DUSP1 in *JAK2* V617F^+^ HEL cells and patients’ bone marrow sections along progression of PV, a factor that was previously shown to be overexpressed also in mouse *JAK2* V617F^+^ BaF3 cells [68]. Additionally, small interfering RNA (siRNA)-mediated knockdown and pharmacological inhibition of DUSP1 led to JNK/p38MAPK reactivation and accumulation of DNA damage, marked by nuclear γH2AX foci accumulation and accelerated cell cycle arrest and apoptosis of *JAK2* V617F^+^ HEL cells, suggesting dependency on DUSP1 for ongoing cell proliferation and survival [82]. Thus, it seems that cooperation of hyper-activated ROS-buffering system and overexpression of DUSP1 represents an “in-built” feature of the *JAK2* V617F oncogene-induced cell rewiring. These mechanisms consequently protect PV progenitors from overall impact of the inflammatory-mediated DNA damage and likely contribute to increased fitness and chronic proliferation of *JAK2* V617F^+^ cells in patients with PV (Figure 4). This model also provides a potential platform for design of novel synthetically lethal therapeutic strategies exploiting *JAK2* V617F-mediated protection mechanisms in chronic phase of PV. Thus, JNK/p38MAPK reactivation by inhibition of DUSP1 may provide an early intervention for elimination of the cycling *JAK2* V617F-positive PV progenitors. Furthermore, simultaneous targeting of essential ROS buffering system components and thereby escalating oxidative stress could then lead to a robust build-up of lethal DNA damage levels, inducing cell death.

## 3. Conclusions

Accumulating DNA damage and chronic inflammation have been suggested as causes for increasing cancer incidence with aging, including incidence of chronic myeloproliferative neoplasms. However, the rate of rise in incidence of *BCR-ABL*-positive CML with advancing age is less prominent than in *JAK2* V617F-positive MPNs [98]. This is consistent with the finding that *JAK2* V617F, albeit at low allelic burden, has been detected commonly in aged, healthy individuals, along with clonal hematopoiesis, and has been considered as one of the pre-leukemia-associated mutations [99,100,101,102,103]. As aging in general is associated with increased inflammation, DNA damage, and senescence-associated secretory phenotype, with myeloid skewing in the hematopoietic compartment [104,105]; this likely provides an environment that selects for the expansion of genetic variants such as *JAK2* V617F [9]. Consequently, age-related clonal hematopoiesis harboring somatic mutations frequently detected in hematologic malignancies is very common, if not inevitable [106].

Acquisition of *JAK2* V617F, in particular, seems to genetically “fix” the already hyperactive JAK2-signaling present in age-associated stressed hematopoiesis [106]. It is also possible that MPN develops as a result of an inflammatory response to the *JAK2* V617F clone that occurs frequently in elderly individuals [107].

These data are in accord with the experimental and clinical evidence that *BCR-ABL* and *JAK2* V617F oncogenes in CML and PV, respectively, have different transforming properties. In contrast to highly transforming potential of *BCR-ABL*, the long latency of chronic indolent phase of *JAK2* V617F-positive MPN (with PV being a good example), where the frequency of transformation is relatively low, supports a model in which PV progenitors avoid detrimental effects of oncogenic and oxidative stress and rather utilize signaling adaptations to extrinsic and intrinsic stresses to prevent accumulation of DNA damage. On the basis of our studies and those of others, we conclude that this fail-safe mechanism against inflammatory stress and DNA damage is, at least partially, mediated by direct JAK2 V617F targets, *RECQL5* and *DUSP1*. Both these molecules might provide candidate therapeutic targets, a functionally defined vulnerability targetable in future attempts to provide early intervention, at the chronic proliferative stage of MPNs.

## Supplementary Materials

The following is available online at https://www.mdpi.com/2072-6694/12/4/903/s1, Table S1: Characteristics of patients at the time of sample collection used for Western blotting (depicted in Figure 1b) and immunohistochemical staining (depicted in Figure 2).

## Appendix A

### Appendix A.1. Methodology for Obtaining Previously Unpublished Data

Electrophoretic mobility shift for Chk2 phosphorylation assay, detection of phospho-Chk1. The pellets of CML and PV patients’ leukocytes obtained from heparinized peripheral blood after erythrocyte lysis were washed with ice-cold PBS and lysed in IP buffer with phosphatase and protease inhibitors. As a positive control for phospho-Chk2 detection by electrophoretic mobility shift, HT-29 cells were irradiated with 6 Gy and before lysis cultured for another 1 or 3 h in RPMI 1640 (supplemented with 10% FBS and 1% penicillin/streptomycin). Protein lysates were homogenized by vortexing and cleared by centrifugation, and 50 µg of protein per well was subjected to Western blot analysis (10% SDS-PAGE gels) with immunodetection using anti-Chk2 (Merck/Sigma, C9108, 1:500), anti-Chk1 (Santa Cruz Biotechnology, G-4, 1:1000), anti-phospho-Chk1 (Ser345, Cell Signaling Technology (CST), 133D3, 1:200), and anti-actin (Merck/Sigma, 1:1000) primary antibodies, followed by a secondary antibody labeled with peroxidase (CST, 1:1000). Cold acetone precipitation was used to increase the protein concentration in almost all patients’ protein samples.

### Appendix A.2. Patients

The present study included 19 patients (12 in CP-CML; 7 with PV); results from 16 patients are included in Figure 1b and Figure 2, and these patients’ characteristics are summarized in Supplementary Table S1. The study by Stetka et al. [82] included 8 PV patients, 14 samples from PV with myelofibrosis grade ½, and 9 samples from post-PV MF patients; values of both studies contributed to the pathogenic model proposed in Figure 4. Future larger studies involving more patients are warranted to validate this concept.

All participants signed informed consent form to this study which was approved by the Ethics Committee of University Hospital Olomouc, Czech Republic. The approval code from the ethical committee is “P301/12/1503” and the date is 31 March 2011.
