# Supplementary material for: Role of DNA Damage Response in Suppressing Malignant Progression of Chronic Myeloid Leukemia and Polycythemia Vera: Impact of Different Oncogenes
Source: Cancers (Basel). 2020 Apr 7;12(4):903. doi: 10.3390/cancers12040903 (PMC7226398; doi:10.3390/cancers12040903)
Supplement: Supplementary file 1 [file cancers-12-00903-s001.zip › Supplementary.docx]

Supplementary Material: Role of DNA Damage Response in Suppressing Malignant Progression of Chronic Myeloid Leukemia and Polycythemia Vera: Impact of Different Oncogenes

Jan Stetka, Jan Gursky, Julie Liñan Velasquez, Renata Mojzikova, Pavla Vyhlidalova, Lucia Vrablova, Jiri Bartek and Vladimir Divoky

**Supplementary Table S1.** Characteristics of patients at the time of sample collection used for Western blotting and immunohistochemical staining.

| **Patient** | **Age** | **Diagnosis** | **Months from the time of diagnosis** | **Stage of the disease (CML)** | **RQ-PCR in CML:**  **BCR-ABL/ABL**  **Allelic burden in PV: JAK2V617F** | **Therapy at the time of sample collection** |
| --- | --- | --- | --- | --- | --- | --- |
| **1.** | 76 | CML | 0 | CP | 53.9% | N/A |
| **2.** | 67 | CML | 121 | CP | n.a. | nilotinib |
| **3.** | 62 | CML | 77 | CP | 48.4% | nilotinib |
| **4.** | 57 | CML | 92 | CP | 7.9% | nilotinib |
| **5.** | 73 | CML | 69 | CP | 27.8% | nilotinib |
| **6.** | 81 | CML | 0 | CP | n.a. | N/A |
| **7.** | 54 | CML | 0 | CP | 100% | N/A |
| **8.** | 65 | CML | 18 | CP | negative nested  RT-PCR | imatinib |
| **9.** | 63 | PV | 56 | N/A | n.a. | none |
| **10.** | 38 | PV | 18 | N/A | n.a. | Interferon-alpha |
| **11.** | 43 | PV | 25 | N/A | n.a. | Interferon-alpha |
| **12.** | 63 | PV | 0 | N/A | 47% | none |
| **13.** | 51 | PV with light fibrosis | 0 | N/A | n.a. | none |
| **14.** | 63 | Post-PV MF | 144 | N/A | n.a. | none |
| **15.** | 68 | CML | 0 | CP | 44,5% | N/A |
| **16.** | 14 | CML | 0 | CP | 51,6% | N/A |

CML—Chronic myeloid leukemia, CP—chronic phase; PV—Polycythemia vera, N/A—not applicable, n.a.—not available from the time of sample collection. None of the patients had additional chromosomal abnormalities at the time of sample collection. Nos. 1, 6, 7, 12, 13, 15 and 16 were newly diagnosed patients. All participants signed informed consent form to this study which was approved by the Ethics Committee of University Hospital Olomouc, Czech Republic.
