# Supplementary material for: Role of DNA Damage Response in Suppressing Malignant Progression of Chronic Myeloid Leukemia and Polycythemia Vera: Impact of Different Oncogenes
Source: Cancers (Basel). 2020 Apr 7;12(4):903. doi: 10.3390/cancers12040903 (PMC7226398; doi:10.3390/cancers12040903)

**Supplementary Figure 1. Original images of representative western blots in Figure 1b.**

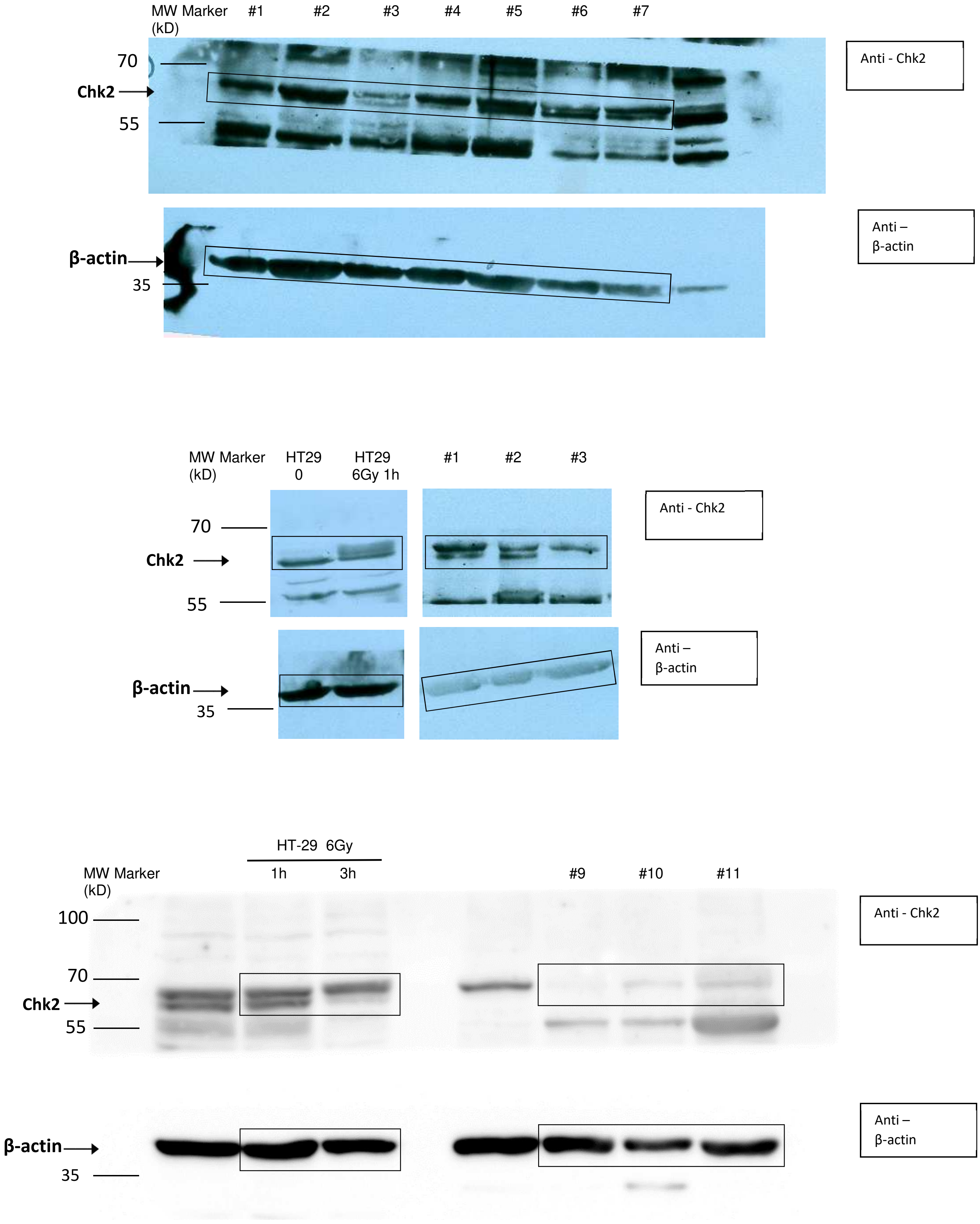

**Supplementary Figure 2. Original images of representative western blots in Figure 1c.**

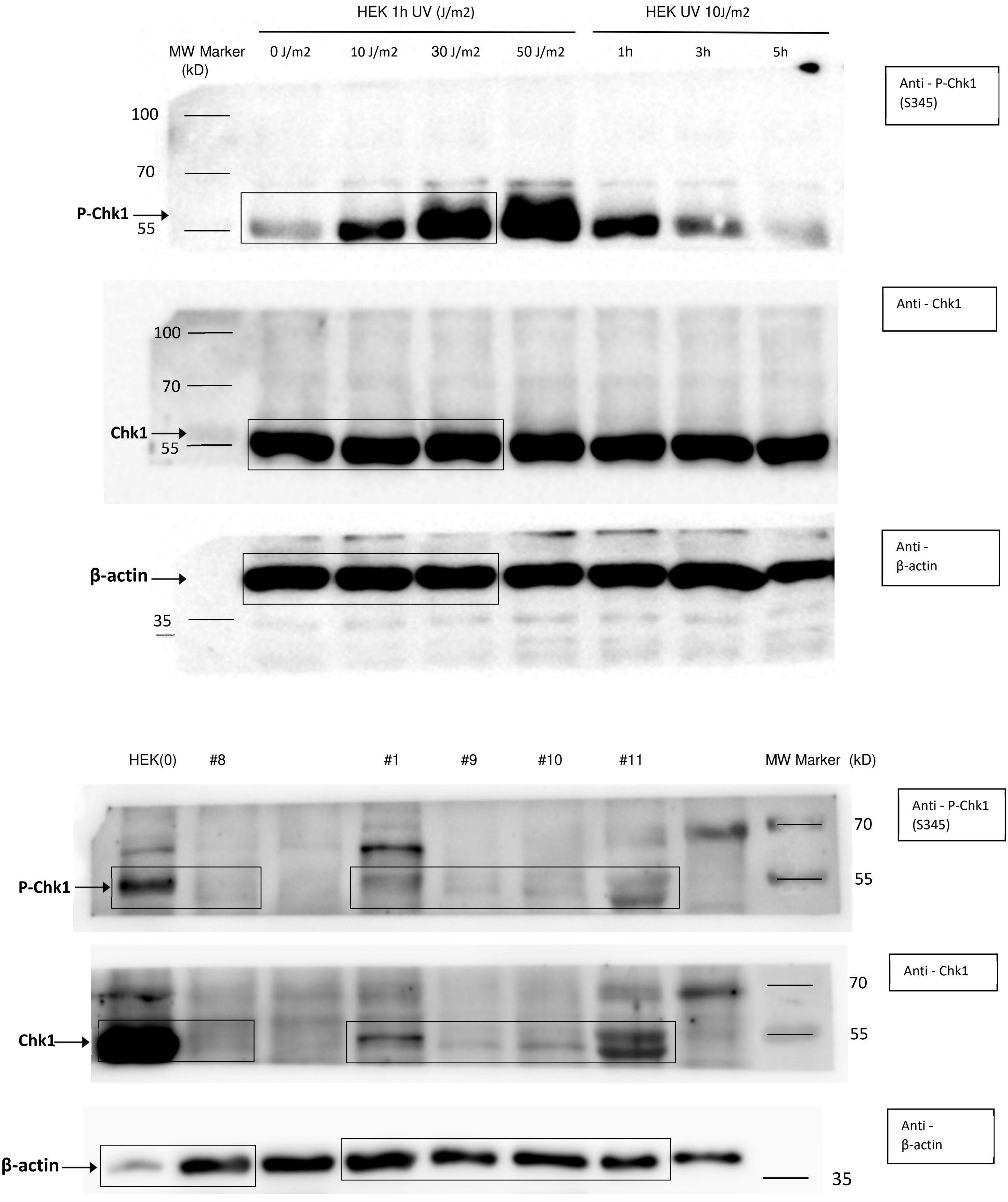

Supplement: Supplementary file 1 [file cancers-12-00903-s001.zip › Uncropped Western blot figures.pdf]
